# Tendinopathy: Pathophysiology, Therapeutic Options, and Role of Nutraceutics. A Narrative Literature Review

**DOI:** 10.3390/medicina55080447

**Published:** 2019-08-07

**Authors:** Carlo Loiacono, Stefano Palermi, Bruno Massa, Immacolata Belviso, Veronica Romano, Ada Di Gregorio, Felice Sirico, Anna Maria Sacco

**Affiliations:** Human Anatomy and Sport Medicine Division, Department of Public Health, University of Naples “Federico II”, 80131 Naples, Italy

**Keywords:** tendinopathies, microscopic anatomy of tendons, pharmacological therapies, physical therapies, nutraceutical products

## Abstract

Tendinopathies are very common in general population and a huge number of tendon-related procedures take place annually worldwide, with significant socio-economic repercussions. Numerous treatment options are commonly used for tendon disorders. Besides pharmacological and physical therapy, nutrition could represent an additional tool for preventing and treating this complex pathology that deserve a multidisciplinary approach. In recent years, nutraceutical products are growing up in popularity since these seem to favor the prevention and the healing processes of tendon injuries. This narrative literature review aims to summarize current understanding and the areas of ongoing research about the management of tendinopathies with the help of oral supplementation.

## 1. Introduction and Epidemiology

Tendon disorders are a class of pathology that includes traumatic injuries as well as chronic diseases, such as tendinopathy. They represent some of the most frequent orthopedic diagnosis, accounting for over 30% of all musculoskeletal consultations [1]. Over 30 million human tendon-related procedures take place annually worldwide with significant socio-economic repercussions in terms of lost working hours and economic expenditure [2].

Tendon disorders are very common in sports. Physical activity determines much stress and force on the tendinous part of the muscle-tendon unit, increasing the risk of tendon injury. Around 50% of sport-related injuries are due to overuse conditions and the majority of these involves tendons [3]. The rotator cuff, the long head of the brachial biceps, the extensors and flexors of the wrist, the thigh adductors, the posterior tibial tendon, the patellar tendon, and the Achilles tendon are the most frequently involved anatomical sites [4]. However, any anatomical district may be interested depending on the type of sport. For instance, dancers show higher prevalence of Achilles tendinopathy, while rotator cuff tendinopathy or epicondylitis are common in rowers [5]. Achilles tendinopathy affects approximately 30% of all runners, with an annual incidence of 7–9%. Patellar tendinopathy is common in volleyball (14%), team handball (13%), basketball (12%), and it is a common condition in football/soccer players (2.5%). Sports such as tennis and baseball show 4 times greater risk of shoulder tendinopathy before the age of 45 years compared with controls [6]. Beyond sport activity, other modifiable and not modifiable risk factors are involved in developing tendinopathy, like age and gender. Adolescents seem to be less affected by tendinopathy compared to adults, since there is evidence that age influences tendinopathy [7]. Although there is no clear trend in the prevalence or incidence between male and female athletes, specific tendinopathies have sex as a risk factor [8]. Even occupational exposure, especially that characterized by highly repetitive movements and poor workplace ergonomics, can predispose one to the risk of tendinopathy. In this case, tendinopathy involves almost exclusively the upper extremity, the most common of which is lateral epicondylitis. A prevalence of 3% have been observed, but rates as high as 18% and 41% have also been reported in spine surgeons and coal miners respectively. Obviously, the type of work influences the prevalence of tendinopathy [5]. Also, different pharmacological molecules can play a negative role on tendon tissue biology and represent a risk factor for developing tendinopathy. Among them, corticosteroids, quinolone antibiotics, aromatase inhibitors, and statins as β-Hydroxy β-methylglutaryl-CoA (HMG-CoA) reductase inhibitors are the drugs most frequently associated with alteration of tendon properties [9]. Finally, tendon pathologies can represent the first clinical presentation of various metabolic diseases. In chronic gouty arthritis and hypercholesterolemia, monosodium urate crystal and cholesterol deposition in the tendons can determine low-grade inflammation, which is responsible for tendon degeneration. In patients affected by diabetes, glycation products deteriorate the biological and mechanical functions of tendons and ligaments [10,11,12]. Even adiposity may be a risk factor for tendon disorders because of the increased weight on the load-bearing tendons and systemic dysmetabolic factors [13]. Moreover, some studies have shown a relationship between dysthyroidism and tendon disorders such as rotator cuff tears [14] and spontaneous rupture of the long head of the biceps tendon [15].

## 2. Anatomy and Physiology

The tendons are “mechanical bridges”. They are strong fibrous structures of pearly color that connect the muscles to the bones and have the main function of transforming the force generated by muscle contraction into movement. In addition to this, they are able to absorb external forces in order to limit muscle overloads and act as temporary energy storage devices. Thanks to their proprioceptive properties, the tendons are also of primary importance in postural adjustments. Anatomically they are organized according to a hierarchical scheme. The smallest building blocks of tendon are collagen molecules. These combine to form tropocollagen helixes. A group of five tropocollagen molecules are bound together by intermolecular crosslinks to form a microfibril (also referred as pentafibrils) and more microfibrils, forming together the fibrils, which have diameters ranging from approximately 10 to 500 nm. The latter grouping together will form collagen fibers with the fibers aggregating once again to make up the largest subunit of tendon, the fascicle. Fascicles are visible to the naked eye, with diameters ranging from 150 to 500 μm. A very thin lamina of connective tissue, endotenon, binds the fascicles to make the complete tendon unit. The tendon surface is covered by the epitenon, which is a connective tissue sheath continuous with the endotenon. It facilitates the sliding of the various structural units and holds blood and lymphatic vessels and nerve structures. Finally, in regions far from joints, an additional loose connective tissue layer, the paratenon, surrounds the tendons and facilitates movement of tendons below the skin [16].

Structurally, the tendons consist of a cellular part, and an extracellular matrix. The tenoblasts are immature spindle-shaped cells. With maturation, they take on a more elongated shape and become tenocytes [17]. These two cell types are the most representative cell types, covering 90–95% of the cells of the tendon and represent a particular type of fibroblasts lying between the collagen fibers along the long axis of the tendon. They have the main function to produce Extracellular Matrix (ECM), such as collagen, fibronectin, and proteoglycans, to maintain tendon homeostasis and repair injured tendons. The remaining 5–10% consists of chondrocytes, synovial cells (outer sheath), capillary endothelial cells, and arteriolar smooth muscle cells [18].

The tendon cells are surrounded and supported by the extracellular matrix. The ECM is a complex structure made up of three major classes of biomolecules: structural proteins (collagen and elastin), specialized proteins (e.g., fibrillin and fibronectin), and proteoglycans [19]. Tendon cells and the ECM have a very close and bidirectional connection; alterations of the ECM may be a consequence of cellular signals, as well as changes in the extracellular microenvironment may lead to cell proliferation, migration, apoptosis, and morphogenesis [20,21]. The balance between these two components is essential to ensure tissue homeostasis, and the alterations in the synthesis and degradation of ECM lead to structural deterioration and degeneration of the tendon.

The main component of the ECM is type I collagen, which accounts for 70–80% of the dry weight of the tendon and represents almost 95% of the total collagen. Other collagens including collagen types II, III, IV, V, VI, IX, X, XII, and XIV have also been found in small quantities within tendon. Elastic fibers (2% of dry weight) give the extensibility, allowing the tendon to stretch in the necessary directions. Proteoglycans are protein–polysaccharide complexes (GAGs) that have the ability to retain water and are responsible for the resistance of the tendons to compressive forces [22,23]. In addition to this, they influence other important physiological processes such as ion transport, nutrient diffusion [24], interactions between cells and matrix and sequestration of growth factors and enzymes in the matrix. In vivo studies demonstrate that biglycan may have both a structural and a signaling role [25]. Decorin, fibromodulin, and lumican are fundamental for the regulation of collagen fibrillogenesis. Non-collagenic glycoproteins as fibronectin and tenascin-C are key factors in the tendon repair process by promoting fibroblast migration, and adhesion of fibroblasts to fibrin [26].

## 3. Microscopic and Macroscopic Pathological Changes

The term “tendinopathy” describes a clinical condition characterized by pain, swelling, and functional limitation of the tendon and contiguous anatomical structures. Except for the infrequent tendon ruptures of a traumatic or iatrogenic nature (e.g., corticosteroids, fluoroquinolones), most tendon pathologies are due to overuse conditions both in the working environment and in sport. Histologically in the typical conditions of overuse, around and inside the tendon, it is rarer to find inflammatory processes. Therefore, the term “tendinitis” is not adapted to describe this condition. It has been shown that there is no inflammatory process at the base of the tendinopathies, if not in the very early stages of the disease (first three weeks from the onset). In fact, in most cases, degenerative phenomena are detected rather than inflammatory, and the use of corticosteroids or anti-inflammatory drugs is mostly ineffective [3]. Regular and well-structured exercise has a positive effect on the tendon tissue by strengthening it through the production of new collagen fibers [27]. But exercise is a “double-edge” sword; indeed, while exercise is the cornerstone of the rehabilitation process in tendinopathies, an overload could determine negative effects on them. In fact, when the tendons are overloaded and subjected to repeat stretching, the collagen fibers begin to slide on top of each other, breaking the cross-links and starting the degenerative process [28,29]. Moreover, when the tendon is subjected to strenuous exercise, high temperatures are developed in the inside that can increase the apoptotic phenomena. Thermal stresses and hypoxia may result in over-expression of molecules such as matrix metalloproteinases (MMP-3) that favor the degradation of the matrix, and the overproduction of inflammatory cytokines such as platelet derived growth factor (PDGF), leukotrienes, and prostaglandin E2 (PGE2). Moreover, hypoxia is associated with an increased expression of vascular endothelial growth factor (VEGF) that promotes neo-angiogenesis and is able to up-regulate the expression of MMP proteins and down-regulate the tissue inhibitor of metalloproteinase-3 (TIMP-3) thus making itself responsible for alteration of the mechanical properties of the tendons. When the overload exceeds the recovery capacity of the tissue or an adequate recovery period is not respected, the repair mechanisms are lost and the pathogenetic cascade leading to tendinopathy occurs [13].

Although it is possible to find inflammatory cells, the most represented histological aspect is the degenerative one. Microscopically the collagen fibers lose their parallel arrangement, appear disorganized and there is increased extracellular matrix [26].

At the level of the extracellular matrix, it is frequently observable mucoid material with a simultaneous separation of the collagen fibers. Degenerate fibers can be replaced by calcification or lipid infiltrates that give rise to the well-known phenomenon of tendolipomatosis. Therefore, there is a marked increase of type III collagen which, compared to type I collagen, is characterized by a lower number of cross-links between and within the tropocollagen units. The clinical relevance of the various degenerative aspects (mucoid degeneration, tendolipomatosis, and calcific tendinopathy) remains uncertain: either alone or in combination, they are also found in a high percentage of healthy and asymptomatic individuals over the age of 35 [13]. In addition to degenerative aspects, in tendinopathies it is of small blood vessels and ingrowths of small nerves. Structural alterations of tendinopathies are also reflected in different imaging techniques, primarily ultrasound. Normal tendon is characterized by uniform alignment of fibers. Tendon texture appears homogenous with parallel echogenic lines reflecting the internal fibrillar structure of the tendon. In tendinopathy, the fibrillary disorganization and the lack of parallel aligned fibers generate multiples reflections and shadowing that are represented by an area of hypoechogenecity. Neovascularization within the pathological tendon is another common finding that can be imaged with color and power Doppler ultrasound imaging. The presence of new vessels can be observed in the hypoechoic areas (matrix disorganization) suggesting that the infiltration of blood vessels may be opportunistic [30].

## 4. Common Therapeutic Options

Numerous treatment options are proposed in the treatment of patients affected by tendinopathies. Usually, they can be grouped in pharmacological therapies, physical therapies, and therapeutic exercise.

Pharmacological treatment of tendinopathies is difficult. Anatomically, these structures suffer from a reduced vascularization and therefore drug availability in the target tissue is low. Moreover, while the effectiveness of drugs on other components of the musculoskeletal system has been proved, such as bisphosphonates in bone [31], myorelaxants in muscle [32], and anticonvulsants in peripheral nerve diseases [33], no specific tendon-target drugs have been developed.

For these reasons, painkillers administrated orally and/or parenterally are often used in the treatment of tendinopathies. NSAIDs (non-steroidal anti-inflammatory drugs), SAIDs (steroidal anti-inflammatory drugs), and other alternative injective approaches have been used.

NSAIDs, in the absence of a manifest inflammatory process, do not change the course of chronic tendinopathy [4] and can have negative effects on the regenerative processes of tissue and should be used with caution [34]. Corticosteroids still represent an extremely common treatment. Many experimental studies suggest that the action of corticosteroids on tendon tissue is harmful. Fibrillary organization loss, reduction of cellular vitality, and tendon derived stem cells (TDSCs) cellular pooling and worsening of the mechanical properties of the tendon tissue have been observed [35]. Some meta-analysis showed that corticosteroid could have a short-term effect on pain in some tendinopathy, like lateral epicondylitis [36], but their long-term use seems not more effective than other treatment options and lacks a pathophysiological rational. Hyaluronic acid (HA) has been shown to have anti-inflammatory and positive effects on cell proliferation and collagen synthesis [37]. The use of low molecular weight hyaluronic acid has proven to be an effective tool in the management of various tendinopathies such as epicondylitis [38] and patellar tendinopathy [39], but the literature is still limited. Platelet-enriched plasma (PRP) is a bioactive component of blood and appears to stimulate tissue repair processes through the activation of chemotaxis, proliferative, and anabolic cell responses. In vitro and animal model studies showed positive effects in terms of cell proliferation, collagen synthesis, and wound healing processes while fewer convincing results were found in human studies probably due to the use of different preparations and protocols (number and frequency of sessions) and the low number of randomized controlled trials (RCTs) [40]. Prolotherapy is an injection therapy consisting of repeated injections of different solutions, generally concentrated dextrose, D-glucose or phenol-glycerine-glucose (P2G), at or near the site of connective tissue dysfunction. These molecules cause an inflammatory reaction at the injection site followed by fibroblast proliferation and collagen deposition [41]. Despite encouraging results in the treatment of different tendinopathies (lateral epicondylitis, Achilles tendinopathy, rotator cuff tendinopathy, and plantar fasciitis), the interpretation of positive results is limited by methodological shortcomings, including small sample size, inadequate control groups, and lack of blinding [42].

Other therapeutic options exist (like trinitrine glycerine patches) but are less used and not available in several countries: among them, only a few have been tested in clinic (i.e., dry needling [43] or high volume image guided injection [44]).

Several physical therapy approaches have been developed during the last years. ESWT (Extra-corporeal shock wave therapy) has become popular in recent years for the treatment of soft tissue disorders, including calcific tendinopathies of the rotator cuff, epicondylitis, plantar fasciitis, Achilles and patellar tendinopathy. It seems they promote the differentiation of tendon derived stem cells (TDSCs), the proliferation of tenocytes and the synthesis of collagen. Moreover, they reduce the production of interleukins and metalloproteases in damaged tendons [45] and determine the release of substances that inhibit pain (endorphins), the induction of specific growth factors such as insulin-like growth factor 1 (IGF-1) and transforming growth factor beta 1 (TGF-β1) which have a mitogenic and anabolic role and increase blood flow [46]. PEMFs (Pulsed electromagnetic fields) are part of the biophysical stimulation methods [47,48,49], but results reported in literature about it are often ambiguous and conflicting [32,33,34]. EPI (intratissue percutaneous electrolysis) technique uses a flow of cathodic current directed to the area of degenerated tendon through an ultrasound-guided needle and determines a localized organic reaction that leads to regenerative phenomena [46]. Some studies have shown encouraging results in the treatment of patellar tendinopathy [50], epicondylitis [51], and proximal hamstring tendinopathy-related sciatic nerve entrapment [52].

Irrefutably, a cornerstone in the treatment of tendinopathy is represented by therapeutic exercise. Several protocols of exercise have been proposed and investigated. Although the exact mechanism is not still completely understood, eccentric exercise with lengthening of muscle during contraction has had a major role in the treatment of tendinopathy. Indeed, it seems to be very effective, especially in the management of patients affected by tendinopathies of the Achilles and patellar tendon. These exercises have been shown to promote cross-linking of collagen fibers and facilitate tendon remodeling [4] and cause an upregulation of insulin-like growth factor (IGF) that promotes cellular proliferation and matrix remodeling [46]. Mechanical proprieties of physical exercises are able to affect tendon’s fibers reorganization and healing [53]. Some protocols include eccentric exercise alone, such as Alfredson protocol for Achilleous tendinopathy [54]. Other protocols like Stanish et al. [55] permitted concentric contraction and stretching also. Some other approaches, like heavy slow resistance training [56] required the application of specific instrumentations to enhance the load on tendinous structures.

No single protocols have shown a definite superiority versus other protocols and the adoption of several protocols of exercise, including eccentric exercise, seems to guide the rehabilitation process, as load is able to stimulate tissue regeneration and reorganization. Doubts about volume, frequencies, velocity, and pain perception during exercise still remain [4].

Many other types of treatment for tendinopathies have been described in the literature but no single therapy has been shown to be more effective than others and often the same technique has shown ambiguous results. The common denominator of the different approaches proposed in the literature is to promote a regenerative response of the tendinous tissue. In fact, due to new initiatives in the pathophysiology of tendon injuries, the use of therapies with anti-inflammatory effect should be abandoned in favor of strategies that promote tissue regeneration.

## 5. Nutrition

In addition to pharmacological and physical therapy, nutrition could represent an effective tool for preventing and treating a pathology that requires, due to its complexity, a multidisciplinary approach. The use of nutritional supplements should be carefully taken into consideration, due to their metabolic effects; for example, the abuse of anabolic-androgenic steroids shows an increased risk of upper body tendon rupture [57]. Particularly, this aspect is even more important for professional athletes, for whom some substances could violate the doping regulamentation and be considered illegal [58].

Some metabolic pathological diet-related conditions could affect tendon function. For example, hypercholesterolemia is a known risk factor for the development of tendinopathy and is a clinical finding frequently seen in cases of rotator cuff tear and Achilles tendon rupture. Furthermore, the management of hypercholesterolemia through statin therapy has shown positive effects on tendon healing [59]. Diabetes mellitus is accompanied by an alteration of the expression of angiogenetic and growth factors as well as of the levels of inflammation mediators and collagen synthesis and its association with tendon diseases is well known [60]; in literature there are strong evidences that diabetes is associated with higher risk of tendinopathy [61], such as Achilles tendinopathy [62] and rotator cuff tendinopathy [63].

Diet plays a primary role in the homeostasis of all tissues and it is an important component in the etiology and management of various medical conditions that can contribute to the development of tendon pathology. The role of dietary supplementation on tissue function and architecture is not a recent topic of research; indeed, in past years, several studies [64,65] have tried to investigate this relationship, but nowadays some nutrients have received more attention and diffusion in the general population.

In general, nutrients can be classified into three categories: macronutrients, micronutrients, and water.

Water is essential for life and it represents a critical nutrient whose absence would be quickly lethal [66]; its importance in the elasticity of aging connective tissue (i.e., tendon elasticity) has been studied in literature [67].

The former consists of carbohydrates, lipids, and proteins. Carbohydrates are the main source of energy that is mainly used for muscle and nervous system metabolism. Proteins are essential for anabolic and repair processes in the musculoskeletal and other tissues and in hormone synthesis. Lipids are an energy source of storage. Vitamins and minerals are considered micronutrients and are required in smaller quantities. Vitamins act as enzymatic catalysts and regulate the chemical reactions that take place in metabolic processes. Minerals have a structural and metabolic function. For example, the matrix metal proteases (MMPs) are zinc-dependent enzymes that play a key role in the metabolism of tendon tissue. Many of the micronutrients are essential and as such must be integrated through the diet [68].

Vitamin C deficiency is not common in the general population of developed countries but can occur in undernourished and/or digestive problems (alcohol abuse, critically ill patients, chemotherapy etc.). It can lead to scurvy, hemarthrosis, synovitis, and arthralgia. Ascorbic acid is well known for its antioxidant properties and as a cofactor in two critical stages of collagen synthesis: hydroxylation of proline and lysine to hydroxyproline and hydroxylysine [69]. This indicates that a deficiency of vitamin C could determine a reduction in collagen synthesis at the musculoskeletal level [70], as demonstrated by a study that have identified this mechanism as the first reason for some rheumatologic manifestations [71]. In a study conducted on pigs it was shown that vitamin C deficiency leads to a reduction in collagen synthesis at bone, cartilage, and tendon levels [72]. Ascorbic acid is a powerful inducer of collagen synthesis in tendon cells. After an injury, the vitamin levels required for proper healing could be greater than those needed for homeostasis in a balanced situation. In an in vivo animal study, the injection of 150 mg of vitamin C in rats with tendon lesions resulted in a better healing process than controls [73].

Vitamin D, in addition to regulating bone metabolism, also acts directly on the synthesis of collagen by the tenocytes. The addition of vitamin D in fibroblast culture media obtained from human tendons demonstrated a dose-dependent anabolic effect with progressive increase in type I collagen mRNA levels and at the same time a reduction in the reactive species of oxygen and expression of the metal protease of the matrix (MMPs). These data demonstrate a positive effect of this vitamin on tendon tissue, and its deficiency could be a limiting factor for collagen synthesis and cause increased exposure to oxidative stress [74]. In another in vitro study on fibroblasts obtained from ligaments, vitamin D has been shown to inhibit their differentiation into osteoblasts and the formation of calcifications (anatomo-pathological finding commonly found in tendinopathies) [75]. Several epidemiological studies [76,77] have shown a correlation between vitamin D deficiency and tendon injuries. In one of these, for example, 80% of subjects undergoing surgical repair of the rotator cuff were vitamin D deficient [68].

Amino acids are the fundamental units for protein synthesis. Among them a positive relationship between leucine and collagen synthesis has been demonstrated. It is an essential element that is required to be introduced with the diet. Several studies have shown this relationship. In one of these, malnourished mice were subjected to a normal diet (control) and enriched with leucine (intervention). Both groups were further divided according to the level of physical activity to which they were subjected. The main result was the demonstration of a positive effect of the high leucine content diet on collagen synthesis especially in the subgroup subjected to physical exercise. Leucine has led to an increase in hydroxyproline levels which, besides being a fundamental component of collagen, plays an essential role in fiber stability [78]. Glycine is also a fundamental element in collagen synthesis. A study has shown that a diet containing 5% glycine correlates with a greater synthesis of hydroxyproline and glycosaminoglycans and therefore with a greater ability to synthesize collagen molecules. This would result in higher fiber strength and would support the hypothesis that a glycine-enriched diet could be beneficial after tendon injury [79]. Also lysine, proline, and cysteine are important factors in collagen synthesis and their integration, especially for lysine as it is essential, could support recovery from tissue damage.

An uncontrolled diet rich in saturated fatty acids can lead to hypercholesterolemia which, as is known, correlates negatively with tissue homeostasis even at tendon level. The higher proportion of cholesterol is carried within the low density lipoprotein (LDL). The oxidized form (oxLDL) has a high pathogenetic potential. A previous research [80] have shown that a diet rich in saturated fats causes significant metabolic and ultrastructural tendon alterations in murine models. It has been shown that oxLDL results in cellular apoptosis, reduced expression of COL1A1 and COL3A1 (genes for collagen I and III), and over-expression of the metal protease of the MMP2 matrix [81].

Some of the most common supplements proposed to preserve and improve the function of connective tissue, are represented by glucosamine and chondroitin sulfate. Several in vivo and in vitro studies [82,83] have shown positive effects of these supplements on tendons as well. Tenocytes cultured in a medium enriched with glucosamine and chondroitin sulfate showed a significantly higher collagen protein synthesis than the controls (+22%) [84]. In an in vivo study on mice subjected to tenotomy, the group receiving a diet enriched with glucosamine and chondroitin sulfate showed a better ultrastructural organization of the collagen fascicles and less inflammation than the control group. Furthermore, at eight weeks, the intervention group showed greater tendon resistance and better biomechanical properties, probably as a consequence of the lower tissue inflammation and greater collagen synthesis [85].

Curcumin is a powerful antioxidant extracted from curcuma longa that shows interesting characteristics. In addition to its antioxidant potential, it has shown positive effects on cell regeneration, wound healing and negative effects on neo-angiogenesis (anatomo-pathological finding frequently found in tendinopathies). In addition, inhibition activity was observed with respect to the matrix metal proteases (MMPs) [86]. In an in vivo animal study, the effect of oral supplementation with curcumin (100 mg/kg) on mice with patellar ligament injury was evaluated. The histological study of the animals treated showed a better organization of collagen fibers, improvement of biomechanical properties and an increase in manganese superoxide dismutase (MnSOD) activity [87].

Since the 1960s, several studies [88,89] have shown that bromelain has positive effects in the treatment of edema and ecchymosis in post-trauma. This substance is a complex of proteases derived from pineapple that has shown different therapeutic effects in many inflammatory diseases. In vitro studies conducted on mouse models have shown a positive effect on tenoblast proliferation and on malondialdehyde (MDA) levels, markers of oxidative stress [90].

There are two other substances that often appear in nutraceutical preparations, boswelic acid and methylsulfonylmethane, of which an anti-inflammatory activity has been demonstrated and with positive effects on pain in muscle and tendon disorders [86,91,92]. Indeed, due to its ability to permeate membranes and penetrate throughout the body, the mechanistic function of methylsulfonylmethane involves a collection of cell types; moreover, studies suggest that this substance operates at the crosstalk of inflammation and oxidative stress at subcellular level [92]. The pharmacological effects of boswelic acid were mainly attributed to suppression of leukotriene formation and migration via inhibition of 5-lipoxygenase [93].

In conclusion, the effectiveness of oral supplements in the management of tendinopathies have been widely investigated by numerous clinical studies; results tend to confirm preclinical evaluations, but are biased as multiple substances were included in the same formulation and poor methodology was adopted [91,92]. For these reasons, it is not possible to draw any definitive recommendations on the use of nutraceutical supplementation in tendinopathies, and further studies are required to improve our knowledge on this topic. Anyway, many nutraceutics are, at date, commercially available and regularly used in clinic for the management of tendinophaties, anticipating scientific evidences on their pharmacological efficacy on treated patients.

## 6. Conclusions

There are many pathways through which nutrition can positively or negatively affect tendon homeostasis. In recent years, nutraceutical products are multiplying that seem to favor the healing processes of tendon injuries and are likely to play a role as prevention tools. The preclinical results seem encouraging even if the same aspect in literature is still too limited. Indeed, the management of tendinopathies with the aid of oral supplementation is a relatively new approach. A specific evaluation of each substance is difficult, also because most of the time combinations of these elements are used and “multivitamins” combinations are adopted in athletic and general population [94].

Their application could have a role in tendinopathy’s prevention, in the therapeutic management of acute and chronic tendinopathy, and in the rehabilitation phase, during with some results showed improvement in the rehabilitation process [92].

Moreover, nutraceutical supplementation could be used in combination with other therapeutic approach, such as shock wave treatment, with positive effects on pain and function [95].

However, further researches are needed to better understand how these substances interact with cell and tissue biology in order to consciously manage nutraceutics as a therapeutic options for tendinopathies, in which a multidisciplinary approach is mandatory.
